# Energy Absorption Characteristics of CFRP–Aluminum Foam Composite Structure Under High-Velocity Impact: Focusing on Varying Aspect Ratios and Relative Densities

**DOI:** 10.3390/polym17152162

**Published:** 2025-08-07

**Authors:** Jie Ren, Shujie Liu, Jiuhe Wang, Changfang Zhao

**Affiliations:** 1 School of Mechanical Engineering, Nanjing University of Science and Technology, Nanjing 200094, China; liushujie@njust.edu.cn; 2 NORINCO Group Aviation Ammunition Insitute Co., Ltd., Harbin 150000, China; m19254506131@163.com; 3 Department of Engineering Mechanics, CNMM and AML, Tsinghua University, Beijing 100084, China

**Keywords:** composite structure, carbon fiber-reinforced plastic (CFRP), aluminum foam, energy absorption, impact loading, crushing behavior

## Abstract

This study systematically investigates the high-velocity impact response and energy absorption characteristics of carbon fiber-reinforced plastic (CFRP)—aluminum foam (AlF) hybrid composite structures, aiming to address the growing demand for lightweight yet high-performance energy-absorbing materials in aerospace and protective engineering applications. Particular emphasis is placed on elucidating the influence of key geometric and material parameters, including the aspect ratio of the columns and the relative density of the AlF core. Experimental characterization was first performed using a split Hopkinson pressure bar (SHPB) apparatus to evaluate the dynamic compressive behavior of AlF specimens with four different relative densities (i.e., 0.163, 0.245, 0.374, and 0.437). A finite element (FE) model was then developed and rigorously validated against the experimental data, demonstrating excellent agreement in terms of deformation modes and force–displacement responses. Extensive parametric studies based on the validated FE framework revealed that the proposed CFRP-AlF composite structure achieves a balance between specific energy absorption (SEA) and peak crushing force, showing a significant improvement over conventional CFRP or AlF. The confinement effect of CFRP enables AlF to undergo progressive collapse along designated orientations, thereby endowing the CFRP-AlF composite structure with superior impact resistance. These findings provide critical insight for the design of next-generation lightweight protective structures subjected to extreme dynamic loading conditions.

## 1. Introduction

As one of the most traditional and effective energy-absorbing structures, thin-walled metal tubes have been widely used in numerous vehicle collision energy absorption systems [1]. However, with the development of composite materials, carbon fiber-reinforced plastics (CFRPs), as a relatively new lightweight material, have been rapidly introduced into the automotive engineering field due to their high specific energy absorption (SEA) and excellent weight reduction capabilities. CFRP exhibits outstanding performance in terms of stiffness, strength, and crashworthiness [2,3,4]. Aluminum foam (AlF), as a lightweight porous material, possesses excellent acoustic and impact absorption properties and has also garnered significant attention for its superior energy absorption (EA), SEA and weight reduction advantages, showing great potential in the construction and automotive industries. However, due to the high cost of CFRP materials and the limited strength of aluminum alloys, many scholars have proposed and studied metal/polymer hybrid structures to combine the advantages of both [5,6]. Due to their excellent EA and SEA, hybrid structures can be widely applied in various fields such as automotive [7], aerospace [8], and protective equipment for weapons [9].

In recent years, many scholars have conducted numerous experimental studies on metal/polymer hybrid structures, including quasi-static compression tests [10,11,12,13,14,15], impact tests [16,17,18,19,20], and three-point compression tests [21], among others. Significant progress has also been made in understanding the influence of core materials on the EA of hybrid structures. Hou et al. [22] found that polyurethane foam cores play a significant role in improving peak load, failure displacement, and EA. Furthermore, Gan et al. [23] evaluated the EA of several polyurethane foam-filled tapered tubes through deformation modes, force–displacement curves, EA, and SEA, discovering that foam-filled tapered tubes with circular cross-sections far outperform those with square and hexagonal cross-sections in EA. Zhang et al. [24] experimentally studied the crashworthiness of CFRP panels filled with different reinforced core materials under static and dynamic loading, finding that filled specimens exhibited different characteristics in static compression and dynamic impact tests, with aluminum honeycomb being a potential candidate for effective EA under dynamic impact loading. Sun et al. [25,26] investigated the crashworthiness characteristics of circular empty, AlF, and honeycomb-filled CFRP tubes under quasi-static crushing conditions, showing that CFRP tubes filled with lightweight foam and honeycomb generally outperform metals in crashworthiness, demonstrating potential as energy-absorbing structures.

Factors affecting the EA of metal/polymer hybrid structures also include structural design. Xiao et al. [27], combining FE simulations and quasi-static axial compression tests, found that using AlF as a filler significantly improved the stability and impact resistance of CFRP thin-walled square beams. Alia et al. [28] investigated the energy-absorbing characteristics of polymer foams reinforced with small CFRP tubes, showing that the SEA characteristics increase with a decreasing inner diameter-to-thickness (i.e., D/t) ratio. Chen et al. [29] conducted a parametric study on the failure behavior and EA mechanisms of foam-filled CFRP/aluminum alloy hybrid columns under various oblique compression conditions, demonstrating that increasing CFRP thickness significantly enhances the crushing performance of hybrid columns under constrained mass conditions. Yang et al. [30,31] also discussed that increasing wall thickness is more effective than increasing diameter in improving average crushing force and promoting EA. Other scholars have enhanced the performance of hybrid structures by treating composite materials. Jiang et al. [32] improved EA by incorporating Aramid-pulp micro-/nano-fibers as interlayers in CFRP/aluminum honeycomb sandwich materials. Chung et al. [33] enhanced the bending and shear strength of the resulting CFRP/AlF composite structures by subjecting CFRP and AlF panels to oxygen plasma treatment. Additionally, Ilinzeer et al. [34] studied the effects of corrosion on hybrid structures, conducting salt spray tests on different types of hybrid sandwich specimens made of an AlF core layer and CFRP face sheets over various time periods, showing that the average tensile strength and stiffness of the specimens significantly decreased due to corrosion.

Current applications of composite structures in aerospace and weapon protection require adaptation to high-speed and high-load conditions where the mechanical properties of materials can change drastically [35,36]. Additionally, given the stringent size and weight constraints for energy absorbers in aerospace applications, this research investigates the influence of aspect ratio and density on energy absorption performance, providing critical references for the design of buffer energy-absorbing structures. Therefore, in this study, experiments were designed to test the dynamic mechanical properties of AlF materials with different relative densities (the ratio of the mass density of the actual volume occupied by matter to the mass density of the ideal volume enclosed by the outer contour), and FE methods were used to investigate the effects of column aspect ratio and AlF relative density on the mechanical behavior of CFRP-AlF composite structures. Specifically, the dynamic compression properties of AlF with different relative densities were first measured using the split Hopkinson pressure bar (SHPB) system. Then, based on the experimental data, an FE model was established in ABAQUS and validated by comparing it with the experimental results. Finally, a parametric study was conducted using the validated FE model to examine the influence of column aspect ratio (i.e., 9:1, 8:1, 7:1, 6:1, 5:1, 4:1, 3:1, 2:1, 1:1) and AlF relative density (i.e., 0.163, 0.245, 0.374, 0.437) on the EA performance of composite structures. The special novelty herein is that the impact dynamics behavior of CFRP-AlF is further revealed through SHPB testing and FE simulation, and the influence law of aspect ratio and relative density on energy absorption is systematically obtained. These results could provide practical insights into the EA under impact, particularly with regard to protecting heavy-load conditions.

## 2. Experimental Methods

### 2.1. Dynamic Compression of AlF

In accordance with the high-velocity impact conditions, SHPB experiments were conducted to characterize the dynamic mechanical response of AlF under transient deformation. This experimental approach enables quantitative measurement of stress–strain behavior at prescribed impact velocities, providing critical insights into strain-rate sensitivity and energy dissipation mechanisms inherent to the composite structures.

Under the assumptions of SHPB experiments and in accordance with the one-dimensional elastic stress wave theory, the three pulses (incident strain $\varepsilon_{i}(t)$, reflected strain $\varepsilon_{r}(t)$, and transmitted strain $\varepsilon_{t}(t)$) recorded by strain gauges on the incident and transmitted bars exhibit the following relationships, i.e.,

(1) $$v_{1}(t)=C_{0}\left[\varepsilon_{i}(t)-\varepsilon_{r}(t)\right]$$

(2) $$v_{2}(t)=C_{0}\varepsilon_{t}(t)$$

where $v_{1}(t)$ and $v_{2}(t)$ are the left and right interface velocities of the specimen, respectively; *t* is motion time, and $C_{0}$ is the elastic wave velocity of bars.

If the initial thickness of the specimen is $l_{s}$, the strain rate of the specimen (${\dot{\varepsilon }}_{s}$) can be given by

(3) $${\dot{\varepsilon }}_{s}=\frac{v_{1}(t)-v_{2}(t)}{l_{s}}=\frac{C_{0}}{l_{s}}\left[\varepsilon_{i}(t)-\varepsilon_{r}(t)-\varepsilon_{t}(t)\right]$$

After integration, the strain of the specimen is obtained as

(4) $${\dot{\varepsilon }}_{s}=\int_{0}^{t}\dot{\varepsilon }(t)dt=\frac{C_{0}}{l_{s}}\int_{0}^{t}[\varepsilon_{i}(t)-\varepsilon_{r}(t)-\varepsilon_{t}(t)]dt$$

The stress in the specimen can be obtained from the transmitted strain pulse, namely

(5) $$\sigma_{s}(t)=\frac{A}{A_{s}}E\varepsilon_{t}(t)$$

where A is the cross-sectional area of the pressure bar, $A_{s}$ is the initial cross-sectional area of the specimen and E is the elastic modulus of bars. By combining Equations (3)–(5), the uniaxial compressive stress–strain curve of the material at a given strain rate can be obtained. According to the assumption of stress uniformity in the specimen, the following relationship holds, i.e.,

(6) $$\varepsilon_{i}(t)+\varepsilon_{r}(t)=\varepsilon_{t}(t)$$

Of note, the material stress $\sigma_{s}$, strain $\varepsilon_{s}(t)$ and strain rate ${\dot{\varepsilon }}_{s}(t)$ can be derived from any two of $\varepsilon_{i}(t)$, $\varepsilon_{r}(t)$ and $\varepsilon_{t}(t)$.

**Figure 1:**
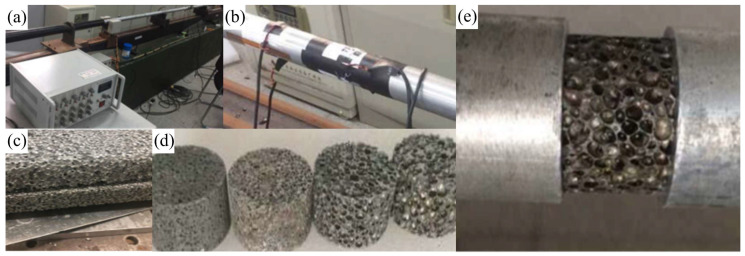
SHPB test system and AlF specimens. (**a**) Data acquisition system, (**b**) schematic of strain gauge installation, (**c**) AlF panel, (**d**) AlF test specimens, and (**e**) specimen installation schematic.

The experimental setup images are presented in Figure 1. The experimental materials consisted of four AlF specimens with different relative densities (i.e., 0.163, 0.245, 0.374, and 0.437), with pore sizes ranging from about 1 mm to 4 mm, having a height of 20 mm and a radius of 16 mm. The impact velocity was controlled at 30 m/s by adjusting the gas source pressure in the launch system. To ensure impedance matching between the incident bar and the test materials, hardened aluminum alloy bars (density is 2.8 g/cm^3^, elastic modulus is 72 GPa, Poisson’s ratio is 0.3) were selected for the SHPB setup. The SHPB system is composed of incident bars measuring 2500 mm in length and transmitted bars measuring 2000 mm in length and a striker with a length of 300 mm, where these bars have a radius of 18.5 mm.

**Figure 2:**
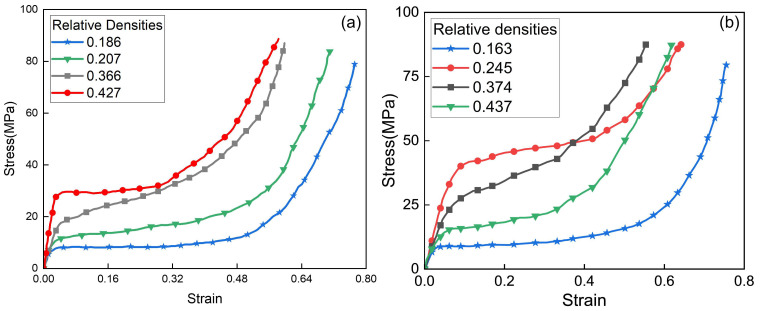
Dynamic stress–strain curves of AlF obtained from SHPB tests. (**a**) $\dot{\varepsilon }=500\;s^{-1}$. (**b**) $\dot{\varepsilon }=1500\;s^{-1}$.

The experimental procedure was as follows: first, the specimen and pulse shaper were installed and clamped between the incident and transmission bars. The high-pressure gas source was then set to the required pressure level; upon releasing the striker bar by opening the valve, the strain signals from the gauges mounted on both bars were recorded by the data acquisition system, all experiments were repeated in triplicate, and the results were averaged; finally, the stress–strain curves of the AlFs were obtained by processing the experimental data in accordance with the aforementioned theories, as shown in Figure 2. The material parameters derived from these tests will be used in subsequent simulation studies.

Stress–strain curves of four types of closed-cell AlF specimens with different relative densities, subjected to varying strain-rate impact conditions, reveal distinct strain-rate dependencies. Specifically, AlF specimens with a higher relative density exhibit greater sensitivity to strain-rate variations, and vice versa. For instance, as shown in Figure 2, for AlF with a relative density of 0.427, both the yield stress and the stress at a given strain increase significantly when the strain rate rises from $500\;s^{-1}$ to $1500\;s^{-1}$. In contrast, specimens with lower relative densities of 0.186 show negligible changes in their stress–strain behavior under the same strain-rate variation.

This strain-rate sensitivity can be attributed to the microstructure of closed-cell AlF: a higher relative density corresponds to lower porosity, causing the material to behave more like the bulk matrix material, which inherently exhibits stronger strain-rate dependence. Conversely, a lower relative density results in higher porosity, making the material structurally closer to a hollow configuration and reducing its resistance to impact loading and responsiveness to strain-rate changes.

### 2.2. Material Properties of CFRP

The CFRP tubes were fabricated using TORAY T700 series carbon fiber prepreg, a widely used aerospace-grade material with balanced strength and stiffness. The preparation process involves wrapping a unidirectional CFRP prepreg around a cylindrical steel mould and then packing it with a vacuum bag. After high-temperature curing, the mould was removed to obtain a long tube, details of which can be found in ref. [37]. The specimens were then produced using CNC machining according to the required size. Section 3.1 explains the ply law and layers of the CFRP laminate tube. The mechanical properties of a single unidirectional CFRP layer were sourced from Ref. [38] and are summarized in Table 1, where the material was regarded to have a two dimensional mechanical state.

### 2.3. EA Characterization Indicators

Commonly used evaluation metrics for the crashworthiness of materials include EA, SEA, average load and initial peak load [11]. SEA refers to the EA per unit mass of the CFRP-AlF composite structure. For the conditions of this study, the following formula for EA can be derived, i.e.,

(7) $$EA=\int_{0}^{x}P\left(x\right)dx$$

and the SEA is calculated as follows:

(8) $$SEA=\frac{EA}{m}$$

where *P* represents the load during the crushing process, *x* is the crushing distance, and *m* is the mass of the crushed portion.

The initial peak load refers to the maximum load experienced during the very short period when the moving body impacts the energy-absorbing structure and the structure just begins to move or deform.

## 3. FE Model

### 3.1. FE Modeling of CFRP-AlF Composite Structure

**Figure 3:**
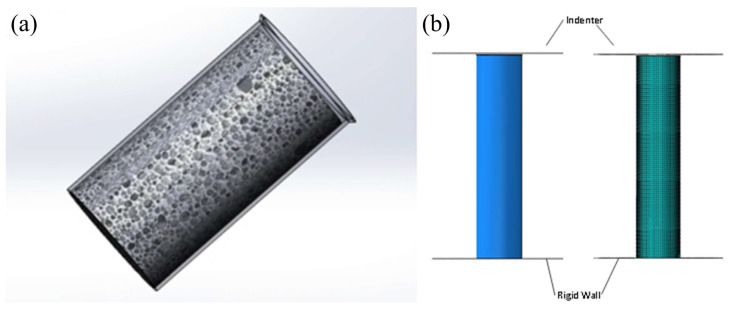
Material and FE models. (**a**) Schematic of CFRP-AlF composite structure, and (**b**) FE model and meshing.

The schematic diagram of the CFRP-AlF composite structure is shown in Figure 3a. To reduce the peak force during impact, a 45° outward chamfer was machined at one end of the CFRP tube. To ensure the chamfer structure functions effectively, the length of the AlF cylinder is slightly shorter than that of the CFRP tube. Figure 3b illustrates the FE model and meshing configuration of the structure. The FE model comprises three primary components: a moving impactor, a fixed rigid wall, and the composite structure. Both the impactor and rigid wall are modeled using discrete rigid elements (element type: R3D4). The impactor with a mass of 200 kg is applied as a concentrated mass at the rigid body reference point. A predefined initial velocity of 30 m/s was applied to the reference point, with all degrees of freedom constrained except for the prescribed impact direction.

The CFRP tube has an inner diameter of 100 mm with a stacking sequence of [45°,0°, − 45°,0°,90°,0°,0°]_2_, totaling 14 plies. Each ply has a thickness of 0.1 mm. Double-layer shell elements were employed to characterize the deformation behavior on both the inner and outer surfaces of the CFRP tube. The two shell layers are connected using cohesive constraints with an offset distance of 0.7 mm (representing the mid-planes of the front and rear seven plies, respectively), and interlaminar strength is *S*_c_. The element type of CFRP is shell, defined as S4R with a mesh size of 2 mm. Considering the high impact velocity, the mass scaling factor was set to 0.98 after multiple trial calculations to ensure computation stability while maintaining solution accuracy.

Based on the experimentally obtained stress–strain curve of AlF, a cylindrical equivalent model was established with a diameter matching the inner diameter of the CFRP tube. The model was constructed using solid elements (element type: C3D8R) with a uniform mesh size of 2 mm. The Crushable Foam material model was employed to characterize its plastic deformation behavior, utilizing volume hardening mode, in which the material parameters, constitutive model and convergence check can be found in Refs. [2,3]. Given the high impact velocity, aluminum foam with a relative density of 0.374 was employed in this simulation to prevent excessive element distortion.

### 3.2. Model Validation

According to the test conditions described in Ref. [11], a FE model was established for a CFRP tube with a 50 mm diameter and 100 mm length. The impactor velocity was set constant at 100 mm/s, with a crushing duration of 0.5 s corresponding to 50 mm crushing displacement.

**Figure 4:**
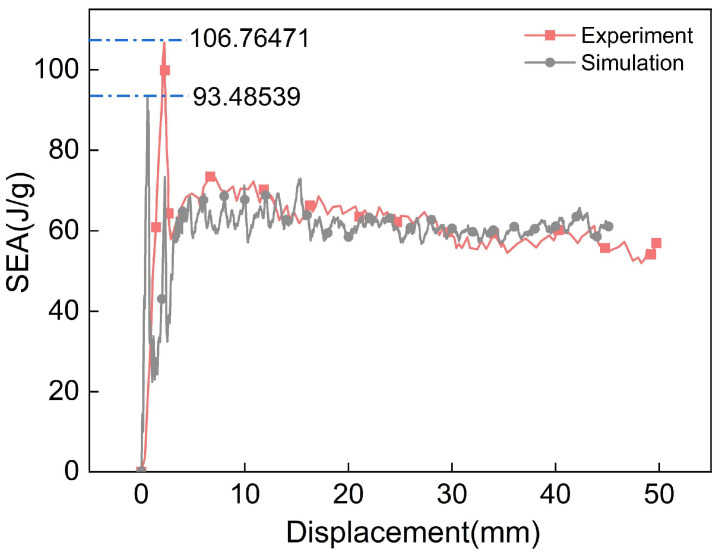
Comparison of SEA-displacement curves between simulation and experiment.

As shown in Figure 4, the SEA curve extracted from the FE model was converted to the same units as those in Ref. [11] and compared with experimental results. Due to the implementation of element deletion in the simulation, the peak value of the simulation appears slightly lower than that of the experimental curve (with a maximum peak difference of 12.4%)**,** with minor fluctuations observed in the latter portion of the curve. Nevertheless, the overall trends demonstrate satisfactory consistency. These observations confirm that the FE model developed in this study can simulate the crushing process of the CFRP tube well.

### 3.3. CFRP-AlF Composite Structures with Varying Aspect Ratios

**Figure 5:**
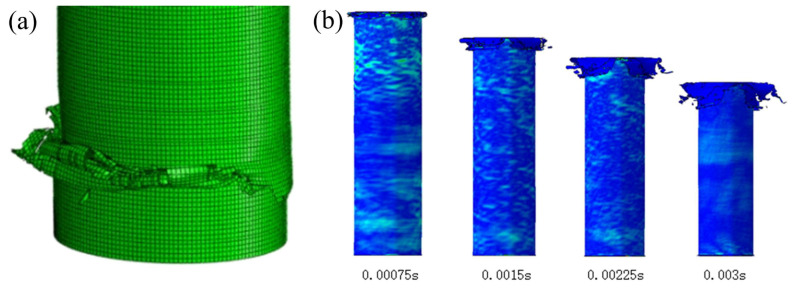
Crushing behavior of CFRP-AlF composite structure. (**a**) Necking fracture, and (**b**) crushing history in CFRP tube.

Considering the structural characteristics, ten filling structures with aspect ratios ranging from 10:1 to 1:1 were selected for simulation, all subjected to an impact velocity of 30 m/s and a crushing stroke of 90 mm, where relative density is fixed as 0.374. Figure 5a illustrates the crushing behavior of the composite structure with 10:1 aspect ratio. It can be observed that necking fracture occurs at the bottom of the CFRP tube immediately upon initial crushing. This necking fracture leads to a significant reduction in both load-bearing capacity and EA of the structure. While the AlF core prevents noticeable lateral deviation of the tube, progressive crushing may eventually cause fracture of the foam column, potentially resulting in substantial structural misalignment and deviation from axial crushing. The observed necking fracture suggests that further increases in aspect ratio beyond 10:1 may exacerbate this failure mode. Consequently, the 10:1 aspect ratio configuration was excluded from subsequent comparative analysis.

Figure 5b presents the crushing sequence of the energy-absorbing structure (using the 5:1 aspect ratio configuration as an example). The impactor initially contacts the CFRP tube, causing immediate crushing and outward curling/tearing deformation at the chamfered end. The presence of the AlF core induces simultaneous outward curling of both inner and outer CFRP layers, demonstrating excellent agreement with the intended deformation mechanism. As crushing progresses, the impactor engages the AlF core, initiating its compressive deformation. The confining effect of the CFRP tube effectively restricts the foam’s deformation to nearly uniaxial compression, thereby optimizing its EA performance.

### 3.4. CFRP-AlF Composite Structures with Varying AlF Relative Densities

Based on experimental data, this study investigates the EA performance of CFRP-AlF composite structures with four different foam relative densities (i.e., 0.163, 0.245, 0.374, and 0.437) under high-speed impact conditions. Following previous findings, the 5:1 aspect ratio configuration was selected for this parametric study due to its favorable deformation mode and moderate plateau stress characteristics. All other simulation parameters remained consistent with the earlier study conditions.

## 4. Result and Discussion

### 4.1. EA Characteristics of Structures with Varying Aspect Ratios

**Figure 6:**
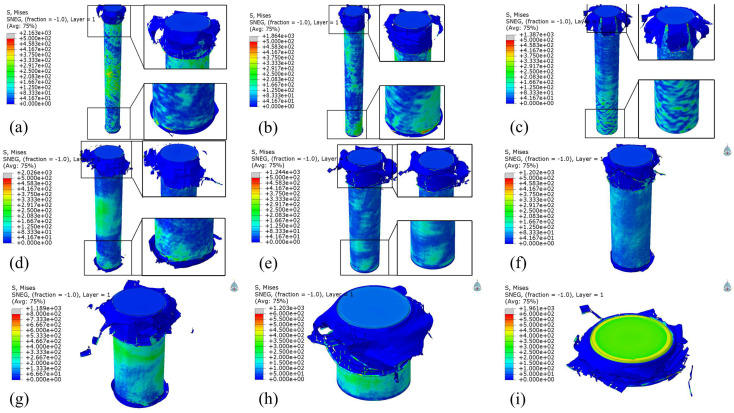
Deformation modes of structures with various aspect ratios including (**a**) 9:1, (**b**) 8:1, (**c**) 7:1, (**d**) 6:1, (**e**) 5:1, (**f**) 4:1, (**g**) 3:1, (**h**) 2:1, and (**i**) 1:1.

Figure 6 shows an isometric view of the deformation patterns for nine structures with different aspect ratios. Various deformation modes can be observed at the impact end, including tearing patterns, “sock-like” folding modes, and minor fragmentation, with generally favorable overall deformation characteristics. For structures with larger aspect ratios, the longer AlF core exhibits lower stiffness and reduced load-bearing capacity, causing the CFRP tubes to bear more of the impact load. This explains why structures with higher aspect ratios show more severe and complex deformation patterns in the CFRP tubes. As the aspect ratio decreases, the deformation modes become simpler, primarily displaying minor fragmentation, interlayer separation, and longer vertical cracks. Additionally, varying degrees of crushing deformation occur at the base of all structures, with only the 7:1 and 5:1 configurations showing minimal base deformation. This is because the simulations did not impose fixed constraints at the base of the material columns. These results demonstrate that in the design of buffer mechanisms, it is essential to implement directional and fixed support measures at the base of the material columns to prevent severe fragmentation, misalignment, and other undesirable phenomena.

**Figure 7:**
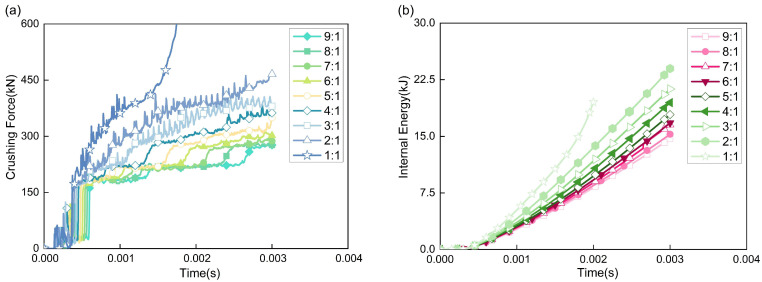
Qualitative results for different aspect ratios. (**a**) Crushing force, and (**b**) internal energy variation.

**Figure 8:**
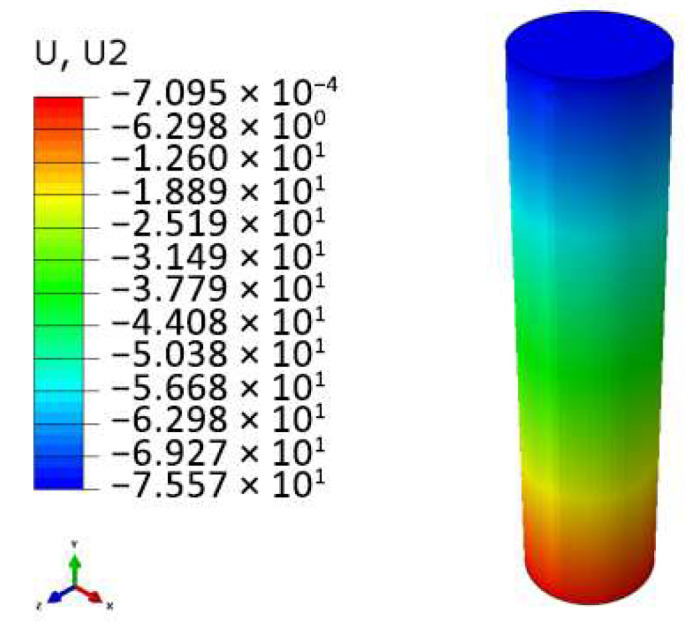
Displacement contour plots of AlF column.

As shown in Figure 7a, the crushing force–time curves of the nine structures reveal that after the impact head contacts the AlF, the foam is gradually compressed until it enters the plateau stage. Except for the 1:1 structure, the plateau stages of structures with other aspect ratios are relatively stable. However, as crushing progresses, the AlF core in structures with larger aspect ratios does not deform uniformly but is compacted in segments, as illustrated by the displacement contour plots of the AlF core in Figure 8. This results in minor step-like fluctuations in the plateau stage. In contrast, structures with smaller aspect ratios have a shorter internal AlF core, which is compacted more rapidly, leading to less noticeable fluctuations and higher average forces in the plateau stage. The 1:1 structure, being compacted the fastest, exhibits a steeply rising segment in the final stage of the crushing force curve, indicating that the material has been fully compacted and the energy-absorbing material is in a rigid collision phase with the impact head. Therefore, only the first two-thirds of its curve are considered. This demonstrates that structures with excessively small aspect ratios are unsuitable for buffer energy-absorbing devices. Additionally, the aspect ratio has little effect on the initial peak load (two peaks) of the impact force, as the two peaks are nearly identical across all curves.

**Figure 9:**
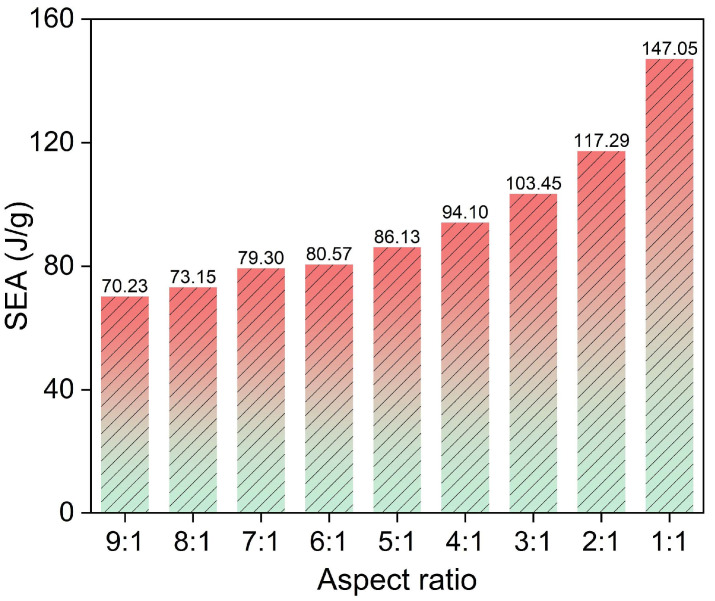
SEA trend for composite structures with different aspect ratios.

As shown in Figure 7b, the internal energy variation trend diagram of the energy-absorbing structure reveals that under identical crushing strokes, the slope of the internal energy–time curve progressively increases as the aspect ratio of the structure decreases (namely from 9:1 to 1:1), which indicates enhanced EA efficiency. However, the accelerated slope growth in the latter segment of the 1:1 aspect ratio structure suggests that smaller aspect ratios lead to faster material compaction, resulting in an earlier rise in crushing force. The SEA values are shown in Table 2, and Figure 9 illustrates the trend of SEA for structures with different aspect ratios. Combining Table 2 and Figure 9, it is evident that as the aspect ratio decreases, the SEA and EA efficiency of the CFRP-AlF composite structures exhibit a quadratic curve trend, gradually increasing. Although the 9:1 and 8:1 structure use more material, their SEA in the initial crushing stage is relatively low, corresponding to lower EA efficiency. The 2:1 and 1:1 structure exhibit higher SEA, but the material is compacted too early, and a significant portion of the absorbed energy results from the rigid collision between the impact head and the compacted material. Consequently, smaller aspect ratios correspond to lower material mass, limiting the total EA. Therefore, aspect ratios between 7:1 and 4:1 are recommended for energy-absorbing devices.

### 4.2. EA Characteristics of Structures with Varying AlF Relative Densities

**Figure 10:**
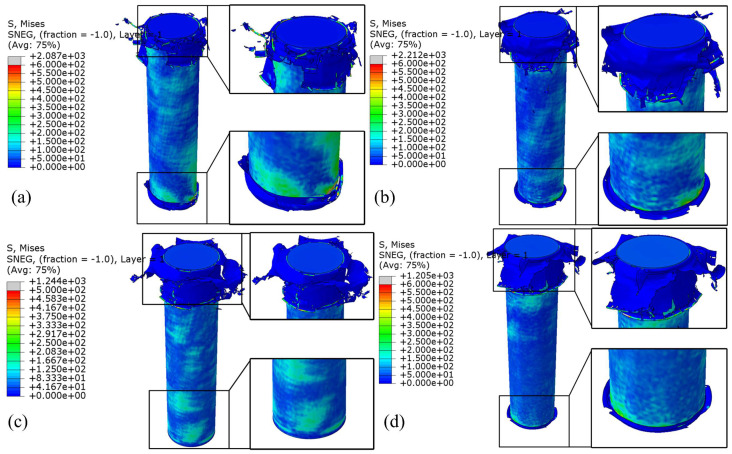
Deformation modes of composite structures with different AlF relative densities including (**a**) 0.163, (**b**) 0.245, (**c**) 0.374, and (**d**) 0.437.

As shown in Figure 10, the deformation modes of structures with different relative densities of AlF core are illustrated. When the relative density is low, it contains more internal pores and weak pore walls buckle sequentially under compression, prolonging the plastic flow regime and delaying densification to higher strains, providing weaker filling and support to the tube walls. Additionally, its yield point is relatively low, resulting in reduced load-bearing capacity. Most of the crushing force acts on the carbon fiber tube, causing severe deformation at both ends. Conversely, when the relative density is high, it contains fewer internal pores, and its mechanical properties align more closely with those of the base material, leading to earlier hardening at lower strain as the load shifts to axial expansion of the metal matrix. The metal column, when crushed, undergoes axial expansion, which can lead to the extrusion of the tube walls. So, in the final stages of crushing, this may cause overall vertical fracture of the tube walls.

**Figure 11:**
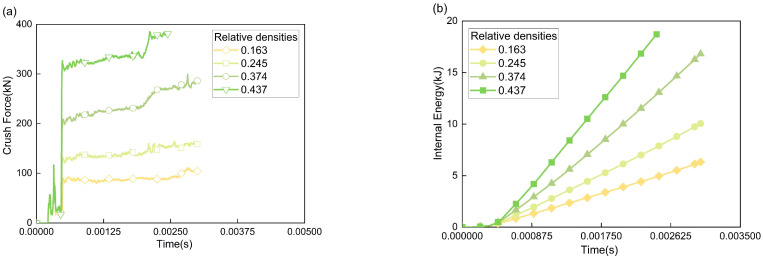
Qualitative results for different relative densities. (**a**) Crushing force, and (**b**) internal energy variation in structures.

As shown in Figure 11a, the force–time curves of structures with different relative densities of AlF core reveal that the two impact peaks remain largely unchanged, and the initial segments of the curves exhibit high overlap. Upon entering the crushing stage, the crushing force experiences a sudden jump, with the magnitude of this jump increasing as the relative density rises. The differences in the plateau stage forces are notably distinct. The curves for relative densities of 0.374 and 0.437 both display a small step-like increase at the end, which occurs because the higher relative density materials, with fewer internal pores, are compacted more rapidly. The material segment first contacted by the impact head is compacted earlier, causing the step to appear sooner compared to the lower relative density materials.

**Figure 12:**
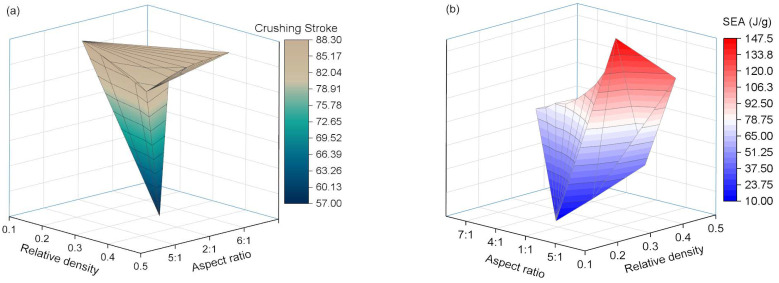
Coupled curve based on relative density and aspect ratio for (**a**) crushing strike, and (**b**) SEA.

As shown in Figure 11b, the internal energy–time curves for energy-absorbing structures with different relative densities of AlF cores indicate that as the relative density increases, both the rate and efficiency of EA by the structure improve significantly. However, due to the substantial magnitude of this improvement and considering the trends in the force curves, the selection of the relative density of the AlF core, in combination with structures of varying aspect ratios, should be carefully considered when choosing energy-absorbing core material. Table 3 presents the EA efficiency of structures with different AlFs relative densities. From the table, it is evident that the AlF core with a relative density of 0.163 exhibits lower SEA and is unsuitable for the high-speed, heavy-load conditions addressed in this study. To investigate the influence of the two key factors (aspect ratio and relative density) on impact crushing further, we plotted a 3D surface map showing the relationship between crushing stroke and SEA, as shown in Figure 12. The analysis reveals that these factors contribute to irregularities in the 3D surface morphology, demonstrating their complex interconnections. This finding highlights the importance of this research in understanding material behaviour under impact conditions.

## 5. Conclusions

In this study, the effects of aspect ratio and relative density of CFRP-AlF composite structures on the EA characteristics were investigated through a validated FE simulation. The main conclusions drawn were as follows: The initial peak load of the CFRP-AlF structures is not significantly affected by the aspect ratio, with the peak loads being relatively close. However, the SEA slightly increases as the aspect ratio decreases. When the length and diameter become increasingly similar, the material is compacted more rapidly. As the relative density of the AlF core increases, the volume occupied by internal pores decreases, the crushing force in the plateau stage increases, and the compaction point occurs earlier. At the same time, the increase in the relative density significantly enhances EA efficiency. Therefore, both EA efficiency and plateau stage forces should be comprehensively considered to appropriately select the energy-absorbing core material. Overall, the CFRP-AlF composite structure demonstrates excellent EA performance and reasonable crushing force levels, while maintaining the advantage of lightweight properties. These characteristics meet the requirements for high-efficiency energy absorption.

The present study on the effects of aspect ratio and aluminum foam relative density on the energy absorption performance of the composite structure can be applied to impact protection applications involving high-speed collisions [39]. In the future, the improvement using different fillers such as polymer foam, fluid medium and even auxetic metamaterials with excellent energy absorption [40] could be further studied. It is expected to provide valuable reference for designing impact energy absorbers in aerospace engineering, particularly for components such as rocket ejection buffer systems.

## Figures and Tables

**Table 1:** Material properties of single unidirectional CFRP layer.

Property	Description	Value
ρ	Density	1.49 g/cm^3^
$E_{1}$	Young’s modulus in longitudinal (fiber) direction	142 GPa
$E_{2}$	Young’s modulus in normal direction (perpendicular to fiber)	8.12 GPa
$G_{12}$	Shear modulus in 1–2 plane	5.95 MPa
*v*_12_	Poisson’s ratio in 1–2 plane	0.021
$X_{T}$	Longitudinal tensile strength (fiber direction)	2.15 GPa
$X_{C}$	Longitudinal compressive strength (fiber direction)	1.43 GPa
$Y_{T}$	Transverse tensile strength (perpendicular to fiber)	50 MPa
$Y_{C}$	Transverse compressive strength (perpendicular to fiber)	196 MPa
*S*_12_	Shear strength in 1–2 plane	82.9 MPa
*S*_c_	Shear strength of interlaminar interface	99.7 MPa

**Table 2:** EA and SEA with different aspect ratios.

Aspect Ratio	9:1	8:1	7:1	6:1	5:1	4:1	3:1	2:1	1:1
Crushing Stroke (mm)	86.00	85.90	85.79	85.65	85.45	85.14	84.69	84.08	57.09
EA (kJ)	14.68	15.27	16.54	16.77	17.88	19.46	21.27	23.98	19.54
SEA (J/g)	70.23	73.15	79.30	80.57	86.13	94.10	103.45	117.29	147.05

**Table 3:** EA and SEA with different AlF relative densities.

Relative Density	0.163	0.245	0.374	0.437
Crushing Stroke (mm)	88.295	87.4755	85.4502	84.4914
EA (kJ)	6.32776	10.0538	17.8818	24.8181
SEA (J/g)	28.3296	45.5180	86.1259	116.6801

## Data Availability

The original contributions presented in this study are included in the article. Further inquiries can be directed to the corresponding author(s).
